# Exploration of self-medication practice in Pokhara valley of Nepal

**DOI:** 10.1186/s12889-020-08860-w

**Published:** 2020-05-19

**Authors:** Sabita Paudel, Bijay Aryal

**Affiliations:** 1 Department of Pharmacology, Gandaki Medical College – Teaching Hospital, Rithepani, Pokhara, Nepal; 2 Karnali Academy of Health Sciences, Jumla Karnali province, Nepal

**Keywords:** Adverse drug reactions, Comedics, Over the counter drugs, Self-medication

## Abstract

**Background:**

Self-medication (SM) is the practice of consuming medication without the consultation of physician. The drugs most commonly self-medicated are paracetamol, analgesics, ranitidine, oral rehydration solution and antibiotics. The objective of the study was to assess the SM status and its causes in Pokhara valley of Nepal.

**Method:**

The study was conducted among the people residing in Pokhara metropolitan city. The study duration was of 4 months from April to July, 2018. The study population were patients attending health general and oral health screening programs at Baidam, Birauta, Hemja and Pame areas of Pokhara. Structured questionnaire was used to collect demographics of the patients and the details of the usage of self-medication.

**Result:**

Out of 201 patients, 38.2% patients were found to be self-medicating. The most common illness sought for SM was ache (headache, body ache) in 50% subjects followed by cough and cold in 31% and gastritis in 23%. Paracetamol was the drug consumed by 16 subjects followed by nimesulide by 11. Lack of knowledge about the disadvantages of SM led to self-medication in 65% of respondents. The personnel most commonly consulted for medication were pharmacists (60%).

**Conclusion:**

The trend of SM is high in Pokhara valley. The comedics were consulted most often for SM due to lack of knowledge of consultation to physicians. The public should be made aware about SM.

## Background

The World Health Organization (WHO) has defined SM as “the use of drugs to treat self-identified symptoms or use of prescribed drug continuously or intermittently for chronic or recurrent diseases without periodic consultation with health care provider” [1]. Most of the government hospitals, relied upon by 3/4th of the country’s population, are not well equipped and unable to fully deliver services to the public. This is contributing highly to the practice of SM by consuming medications with consultation of paramedics [2, 3]. Due to the increment in the literacy rate, people are being aware about the medications; more about over the counter medication. The development of information technology has made people easy access to the internet and smartphones. People can google about their health issues and the medications [3].

Pokhara valley is located in Western part of Nepal. It has 33 wards with population span of 4.2 million. In this metropolitan city, the practice of SM was huge as shown by previous studies [4, 5]. People were self-medicating mainly with non- steroidal anti-inflammatory drugs (NSAIDs). They were consuming these drugs for body ache and headache. The consumption of ranitidine, omeprazole, pantoprazole for gastritis was also large. Consumption of such medications was attributed to self-knowledge, suggestion of relatives and consultation with local comedics [4, 5].

In a Nigerian study, 85% respondents admitted to practice SM. The most commonly self-medicated drugs were analgesics (26.5%) in single and in combination antibiotics, antimalarial and analgesics (15.3%); antibiotics and analgesics (10%) of samples [6]. The practice of SM is overcoming the financial burden in healthcare like reduction of consultation fees, transportation cost. On the other hand, it is accentuating problems of antibiotic resistance, adverse drug reactions and drug interactions. There is a need of strict guidelines regulating patients as well as comedics to ensure optimum utilization of SM practice [7].

This study revealed current status of SM among the study population. In the current Federal system of country, there are seven provinces. Pokhara is headquarter of Gandaki Province. In this initial phase of federalism, there are still confusions in exercise of executive power between central and federal government which is creating difficulties in health sector too [8].

This study would be an important tool for local government to formulate plan of action for the appropriate use of medicines. This study would also be instrumental to the federal government and regulatory agencies to formulate strict rules regarding the use of over the counter and prescription drugs.

This study aimed to explore and assess the status of SM and its causes in Pokhara valley of Nepal.

## Methods

Study design was cross sectional survey.

### Characteristics of participants

There were regular general and oral health screening programs of Bibeksheel Nepali social club in Pokhara valley. The data were collected from four such programs in four regions of Pokhara valley; Baidam, Birauta, Hemja and Pame. Convenience sampling technique was followed. Written informed consent were taken from all subjects**.** All the consenting participants attending screening programs and meeting the inclusion criteria were included. A total of 201 respondents were enrolled. Patients older than 18 years who were able to provide consent were included in the study. Participants with cognitive disabilities with their caretaker or they themselves if able to communicate were included in the study after seeking informed consent.

### Description of methods

Structured validated questionnaire was taken from previous study [4]. It was used to take the data on the particulars of the patient; type of medication consumed among the various groups; duration of consumption of drug, disease/s for which drug/s is/are consumed in last 6 months; person to whom the advice was taken for SM for e.g. self-knowledge or friends or pharmacist and the reasons for not consulting the consultant doctor like lack of knowledge.

### Data entry and analysis

The data were entered in Microsoft Excel and statistical analyses done with Statistical Package for Social Sciences (SPSS) 24.0 version. Chi square test was done to compare between different groups. The level of significance was set at 95% of confidence interval (CI) and *p* value < 0.05 was considered significant. The ethical approval was obtained from the institutional review committee(IRC) of Gandaki Medical College (GMC).

## Result

Among a total of 201 study population, males and females were 44.8 and 55.2% respectively. The age and sex distribution of the study population is presented in Table 1.

**Table 1:** Age range and sex distribution of the study participants

Age Range (Years)	Sex		Total (percent)
	Female	Male	
< 19	1 (0.49)	9 (4.47)	10 (4.97)
20–29	16 (7.96)	21 (10.45)	37 (18.41)
30–39	22 (10.94)	21 (10.45)	43 (21.39)
40–49	18 (8.95)	23 (11.44)	41 (20.40)
50–59	18 (8.95)	15 (7.46)	33 (16.42)
> 60	15 (7.46)	22 (10.94)	37 (18.41)
Total (Percent)	90 (44.78)	111 (55.22)	201 (100.00)

A total of 38.2% were self-medicating in the past 6 months. The age range of subjects with the highest practice of SM was in 30–39 with the significant *P*-value as depicted in Table 2.

**Table 2:** Association of age range with practice of SM

Age Range (Years)	Have you taken self-medication without doctors’ advice in the last 6 months?		Total (%)	*P* value
	No	Yes		
< 19	6 (2.98)	4 (1.99)	10 (4.97)	0.044*
20–29	19 (9.45)	18 (8.95)	37 (18.41)	
30–39	30 (14.92)	13 (6.47)	43 (21.39)	
40–49	31 (15.42)	10 (4.97)	41 (20.40)	
50–59	14 (6.96)	19 (9.45)	33 (16.42)	
> 60	23 (11.44)	14 (6.96)	37 (18.41)	
Total(Percent)	123 (61.19)	78 (38.81)	201 (100.00)	

* Pearson’s Chi-square test

There was no significance of SM practice with the education level (*p* value: 0.068) which is illustrated in Table 3.

**Table 3:** Association of range of education with practice of SM

Range of education	Have you taken self-medication without doctors’ advice in the last 6 months?		Total	*P* value
	No	Yes		
Intermediate and below	106	60	166	0.068*
Bachelors and above	17	18	35	
Total	123	78	201	

*Pearson’s Chi-square test

Only 29 of the participants had known the drug that they had consumed as SM. The most common drug consumed was paracetamol (*n* = 16) followed by nimesulide (*n* = 11). The distribution is shown in Table 4.

**Table 4:** The drugs taken as SM (*n* = 29)

Drugs (if known) used by the respondents	Number
Paracetamol	16
Nimesulide	11
Ibuprofen	6
Ayurvedic oil	6
Ibuprofen+Paracetamol	2
Pantoprazole	2
Ranitidine	2
Cefixime	1
Amoxicillin	1
Azithromycin	1
Topical steroid ointment	1

The most common illness sought for SM was ache (headache, bodyache) in 50% followed by cough and cold (31%) and gastritis (23%) as depicted in Table 5.

**Table 5:** Diseases for which SM were sought (*n* = 78*)

Disease	Frequency of usage	Percent
Ache	39	50.0
Cough and cold	24	30.8
Gastritis	18	23.1
Infection	6	7.7
Hypertension	1	1.3
Others	10	12.8

*Calculations done in the respondents who had sought SM

The comedics (60%) were the paramedics most commonly consulted for medication. They were consulted by 60% subjects. It was followed by self-knowledge in 22% of subjects as illustrated in Table 6.

**Table 6:** Source of advice (*n* = 78)

By whose advise you had taken the medicine?	Number	Percent
Comedics	47	60.3
Self	17	21.8
Health care workers	7	9.0
Others	12	15.4

The lack of knowledge about the disadvantages of SM led to its practice in 65% of respondents as illustrated in Table 7.

**Table 7:** Reason of not consultation to physician

What was the reason of not taking consultation with doctors?	Number	Percent
Lack of knowledge	50	64.1
Financial constraints	7	9.0
Pressure from the family	6	7.7
Others	13	16.7

## Discussion

People were not aware about SM of drugs. They haven’t consulted physician due to lack of knowledge about consultation. So, education is essential. The department of drug administration in Nepal is the authorized body to deal with the drug management systems of the country [9]. It has to regulate the drug distribution system strictly.

The NSAIDs like paracetamol, nimesulide were commonly self-medicated due to ease of availability and the knowledge regarding their use. In many other studies [4–6, 10–12], NSAIDs including paracetamol were the most extensively consumed medication which was similar finding as our study. Whereas in a study in Khartoum state of Sudan, analgesics including paracetamol seconded to antibiotics in terms of consumption [13]. In Ebonyi state of Nigeria, multivitamins and anti-malarial drugs were on top, leaving behind analgesics and antibiotics [14].

The antibiotics like cefixime, amoxicillin and azithromycin were self-medicated in a case each. Lack of knowledge about proper use of antibiotics was leading to antibiotic resistance [5, 10, 15]. It had been an alarming problem in Nepal. WHO has been working in it with strategic plans [16]. The most common diseases include aches and gastritis which corresponds to many other studies done in other part of world [4, 5, 17]. Body ache and headache were the most common presentation of many diseases like influenza, cold, musculoskeletal problems, dysmenorrhea, migraine, etc. [18] The reason of SM of anti-gastritis medication after analgesics was extensive prevalence of gastritis in Nepal [19]. In contrast to our study, in many other studies, the most common illness seeking for SM was fever [4, 5, 11, 12].

The comedics were most often consulted for medicine as they were easily available in pharmacy**.** In many other studies too, comedics were most often consulted for healthcare [4, 11, 13, 20]. Due to the inaccessibility to specialized care and consultants, comedics were sought for treatment of many illnesses. Pharmacies of comedics are easily available next door. In rural areas they were the first person to provide medications in most instances [4].

Prevalence of SM in our study was 38.2% comparable as that of other studies [21, 22]. The high prevalence could be attributed to the easy availability of the drugs over the counter. Also, people could easily know about their health problems due to development of internet services which had reduced visit to physicians. But on the other hand, it has increased tendency of SM [3]. In contrast to our study, practice of SM in urban populations of Ebonyi state of Nigeria [15] was higher. Whereas, in urban Puducherry SM practice was lower than our study [11]. This could be explained on the basis of different regions, health policy and determinants of health.

Other studies had projected the cause of SM practice due to the lack of the knowledge [11, 12]. The education level was not significantly related to SM practice in our study. As our study was done in the urban area, most of the respondents were literate. The recall period of 6 months in our research corresponds to many other similar researches [4, 12] but in Jammu [13] one-year recall period was considered. We chose 6 months to minimize recall bias.

In our study, lack of knowledge about consultation to the physician was the major reason in 65% of subjects. Though education level wasn’t significantly related to SM practice, people expressed their lack of knowledge as the main factor. They weren’t aware about consultation to the general physicians denying fact that symptom could be more serious. Whereas in Nigerian and Srilankan studies, consideration of disease as minor was the main reason [6, 12]. Limitations of this study were sample may not be representative of Pokhara valley and recall bias.

## Conclusion

The practice of SM was high in Pokhara valley of Nepal. Paracetamol was the most extensively self-medicated drug. Headache and body ache were the most common condition seeker for SM. The comedics were consulted most often for SM due to lack of knowledge of consultation to physicians. The public should be made aware about SM. Rampant SM should be strictly monitored.

## Data Availability

The datasets used and/or analyzed during the current study are available from the corresponding author on reasonable request.

## Abbreviations

GMC: Gandaki Medical College
IRC: Institutional Review Committee
NSAIDs: Non-steroidal anti-inflammatory drugs
SM: Self medication
SPSS: Statistical Package for Social Sciences
WHO: World Health Organisation

## References

[1] World Health Organisation (2000). Guidelines for the regulatory assessment of medicinal products for use in self medication.

[2] World Health Organisation. World Health Statistics 2017. Geneva; 2017. https://www.who.int/gho/publications/world_health_statistics/2017/en/. Accessed 20 Jan 2019.

[3] Pradhan MR (2004). ICTs application for better health in Nepal. Kathmandu Univ Med J.

[4] Shankar PR, Partha P, Shenoy N (2002). Self-medication and non-doctor prescription practices in Pokhara valley, Western Nepal: a questionnaire-based study. BMC Fam Pract.

[5] Banerjee I, Sathian B, Gupta RK, Amarendra A, Roy B, Bakthavatchalam P, et al. Self-medication practice among preclinical university students in a medical school from the city of Pokhara, Nepal. Nep Jour Epi. 2016:574–81.. 10.3126/nje.v6i2.15165 PMC5073175 27774346

[6] Omolase CO, Adeleke OE, Afolabi AO, Afolabi OT (2007). Self medication amongst general outpatients in a Nigerian community hospital. Ann Ib Postgrad Med.

[7] Sherazi BA, Mahmood KT, Amin F, Zaka M, Riaz M, Javed A (2012). Prevalence and measure of self medication: a review. J Pharm Sci Res.

[8] Thapa R, Bam K, Tiwari P, Sinha TK, Dahal S (2019). Implementing federalism in the health system of Nepal: opportunities and challenges. Int J Health Policy Manag.

[9] Government of Nepal. Department of Drug Administration: Ministry of Health and population. http://www.dda.gov.np/content/role-of-dda. Accessed 22 May 2019.

[10] Selvaraj K, Kumar SG, Ramalingam A (2014). Prevalence of self-medication practices and its associated factors in urban Puducherry, India. Perspect Clin Res.

[11] dSilva BP, Hussain FH, Ginige G, Kulathinge A, Kannangara H, Goonawardane S (2017). Self-medication practices and misuse of medicine among mothers of young children attending a teaching hospital in Sri Lanka. Sri Lanka J Child Health.

[12] Sharma R, Verma U, Sharma CL, Kapoor B (2005). Self medication among urban population of Jammu City. Ind J Pharmacol.

[13] Awad AI, Eltayeb IB, Capps PA (2006). Self-medication practices in Khartoum state. Sudan Eur J Clin Pharmacol.

[14] Alo C, Oguejiofor NC, Alo NC (2015). Self medication and its pattern among patients attending the general outpatient clinic of a tertiary institution in Abakaliki, Ebonyi state, Nigeria. J Community Med Public Health Care.

[15] Biswas M, Roy MN, Manik MI, Hossain MS, Tapu SM, Moniruzzaman M (2014). Self medicated antibiotics in Bangladesh: a cross-sectional health survey conducted in the Rajshahi City. BMC Public Health.

[16] Resource mobilisation for AMR: Getting AMR into plans and budgets of government and development partners. Nepal country report. https://www.who.int/antimicrobial-resistance/national-action-plans/Nepal-AMR-integration-Report-WHO-Sept-2018.pdf. Accessed 12 Dec 2018.

[17] Suleman S, Ketsela A, Mekonnen Z (2009). Assessment of self-medication practice in Assendabo town, Jimma zone, southwestern Ethiopia. Res Social Ad Pharm.

[18] Colledge NR, Walkar BR, Ralston SH (2010). Davidson's principles and practice of medicine.

[19] Lamichhane DC, Giri BR, Pathak OK, Shankar PR (2006). Morbidity profile and prescribing patterns among outpatients in a teaching hospital in Western Nepal. MJM..

[20] Ramkumar S (2017). Vijayalakshmi1 S, Seetharaman N, Rahman KM. Self-medication practices: an unrealised threat in the country- community based survey from a rural area of Puducherry, South India. Nat’l J Res Community Med.

[21] Limaye D, Limaye V, Fortwengel G, Krause G (2018). Self-medication practices in urban and rural areas of western India: a cross sectional study. Int J Community Med Public Health.

[22] Wijesinghe PR, Jayakody RL, Seneviratne RA (2012). Prevalence and predictors of self medication in a selected urban and rural district of Sri Lanka. WHO South-East Asia J Pub Health.

